# Interactive Compliance Control of a Wrist Rehabilitation Device (WR*e*D) with Enhanced Training Safety

**DOI:** 10.1155/2019/6537848

**Published:** 2019-02-18

**Authors:** Dong Xu, Mingming Zhang, Han Xu, Jianming Fu, Xiaolong Li, Sheng Q. Xie

**Affiliations:** ^1^Auckland Tongji Medical & Rehabilitation Equipment Research Centre, Tongji Zhejiang College, Jiaxing, China; ^2^Department of Biomedical Engineering, Southern University of Science and Technology, Shenzhen, China; ^3^Key Lab of Digital Manufacturing Equipment & Technology, Huazhong University of Science and Technology, Wuhan, China; ^4^Rehabilitation Medical Centre, The Second Hospital of Jiaxing, Jiaxing, China; ^5^School of Electronic and Electrical Engineering, University of Leeds, Leeds, UK; ^6^Institute of Robotics, Faculty of Engineering, Qingdao University of Technology, Qingdao, China

## Abstract

Interaction control plays an important role in rehabilitation devices to ensure training safety and efficacy. Compliance adaptation of interaction is vital for enabling robot movements to better suit the patient's requirements as human joint characteristics vary. This paper proposes an interactive compliance control scheme on a wrist rehabilitation device (WR*e*D) for enhanced training safety and efficacy. This control system consists of a low-level trajectory tracking loop and a high-level admittance loop. Experiments were conducted with zero load and human interaction, respectively. Satisfactory trajectory tracking responses were obtained, with the normalized root mean square deviation (NRMSD) values being 1.08% with zero load and the NRMSD values no greater than 1.4% with real-time disturbance and interaction from human users. Results demonstrate that such an interactive compliance control method can adaptively adjust the range of training motions and encourage active engagement from human users simultaneously. These findings suggest that the proposed control method of the WR*e*D has great potentials for clinical applications due to enhanced training safety and efficacy. Future work will focus on evaluating its efficacy on a large sample of participants.

## 1. Introduction

According to a report from American Heart Association, 33 million stroke cases happened worldwide in 2010, with 16.9 million people having a first stroke [1]. In the United States, more than 700,000 people suffer a stroke each year, and approximately two-thirds of these individuals survive and require rehabilitation [2]. In New Zealand (NZ), there is an estimated 60,000 stroke survivors, and many of them have mobility impairments [3]. Stroke is the third reason for health loss and takes the proportion of 3.9 percent, especially for the group starting on middle age, suffering the stroke as a nonfatal disease in NZ [4]. Stroke is also a serious disease in Europe: 200 to 300 of 100,000 people in Europe suffer from a stroke as new sufferers, and approximately 30% survive with motor deficits [4]. In China, approximately 1.6 million people die of stroke annually, in a total population of 1.4 billion people [5]. In general, stroke has a great adverse impact for many people worldwide, regardless of ethnic groups and regions.

Professor Caplan who studies Neurology at Harvard Medical School describes stroke as a term which is a kind of brain impairment as a result of abnormal blood supply in a portion of the brain [5]. The brain injury is most likely leading to dysfunctions and disabilities. These survivors normally have difficulties in activities of daily living, such as walking, speaking, and understanding, and paralysis or numbness of the human limbs. The goals of rehabilitation are to help survivors become as independent as possible and to attain the best possible quality of life.

Physical therapy is conventionally delivered by the therapist and requires a one-to-one physical therapist to do manual interactions with patients. While this has been demonstrated as an effective way for motor rehabilitation [6], it is time consuming and costly. Treatments manually provided by therapists require to take place in a specific environment (in a hospital or rehabilitation center) and may last several months for enhanced rehabilitation efficacy [7]. A study by Kleim et al. [8] has shown that physical therapy like regular exercises can improve plasticity of a nervous system and then benefits motor enrichment procedures in promoting rehabilitation of brain functional models. It is a truth that physical therapy should be a preferable way to take patients into regular exercises and guided by a physical therapist, but Chang et al. [9] showed that it is a money-consuming scheme. While manual physical therapy plays a crucial role in the recovery from a stroke, it is likely to provide a lack of consistency for objective assessment for determining rehabilitative plans [10]. For this reason, the industry has started to seek more solutions through the manufacture of robotic technologies integrated into the recovery process for supporting or substituting manual therapy.

Robot-assisted rehabilitation solutions, as therapeutic adjuncts to facilitate clinical practice, have been actively researched in the past few decades and provide an overdue transformation of the rehabilitation center from labor-intensive operations to technology-assisted operations [11]. Engineers and people from medical fields are making tremendous efforts to make the rehabilitation robots safer and more adaptable for the human body [12, 13]. The robot could also provide a rich stream of data from built-in sensors to facilitate patient diagnosis, customization of the therapy, and maintenance of patient records. As a popular neurorehabilitation technique, Liao et al. [14] indicated that robot-assisted therapy presents market potential due to quantification and individuation in the therapy session. The quantification of robot-assisted therapy refers that a robot can provide consistent training pattern without fatigue with the given parameter. The characterization of individuation allows therapists to customize a specific training scheme for an individual.

Upper extremity function is of paramount importance to carryout various activities of daily living [15], in which the human wrist plays a vital role when orienting of an object. For the rehabilitation of the human wrist, a variety of robotic devices have been developed in the past few decades [16]. Some rehabilitation robots have been developed by combining the movement of both arm and wrist. The MIT-MANUS has been clinically demonstrated as an excellent tool for shoulder and elbow rehabilitation on stroke patients [17]. Krebs et al. [18] further developed a wrist rehabilitation robot with three rotational degrees-of-freedom (DOFs). This wrist device can be operated stand-alone or mounted at the tip of the MIT-MANUS. Faghihi et al. [19] constructed a three DOFs wrist robot by using a similar structure as the work by Krebs et al. [18]. They only introduced the design and fabrication of the wrist robot without the introduction of a control system. Toth et al. [20] clinically validated the safety and efficacy of the REHAROB in helping spastic hemiparetic patients with passive physiotherapy. This robot was built from two industrial robots, which is not cost effective. Oblak et al. [15] developed a universal haptic device that enables rehabilitation of either arm or wrist movement depending on locking or unlocking of a passive universal joint. Some portable devices were developed with the focus on rehabilitation of the wrist joint. Colombo et al. [21] proposed a single-DOF rehabilitation robot for flexion and extension of the wrist joint. This device is actuated by a direct current motor with the integration of a torque transducer and a potentiometer in providing feedback signals, which allows the implementation of advanced interactive training strategies. Khor et al. [22] developed a single-DOF device that can enable wrist and forearm training in different configurations. This device has not been controlled with advanced interaction modes, while it has advantages of cost effectiveness and portability.

This paper proposes an interactive compliance control scheme on a wrist rehabilitation device (WR*e*D) for enhanced training safety and efficacy. This control system consists of a low-level trajectory tracking loop and a high-level admittance loop. Experiments were conducted with zero load and human interaction, respectively, to evaluate the dynamic performance of the control system. This paper is organized as follows. Following the Introduction, a description of the WR*e*D development is given, as well as its trajectory tracking control system with/without compliance adaptation. The control stability is also analyzed and presented. The section of Experimental Results is introduced next, with the Discussion and Conclusion at last.

## 2. Methods

### 2.1. Wrist Rehabilitation Device (WR*e*D)

The wrist joint anatomically possesses two DOFs: flexion and extension and abduction (radial deviation) and adduction (ulnar deviation) [23]. The WR*e*D presented in this study functions only for wrist flexion and extension rehabilitation training. While this device can be reconfigured into wrist training of radial/ulnar deviation, this does not affect the study design in evaluating the proposed control scheme.

**Figure 1:**
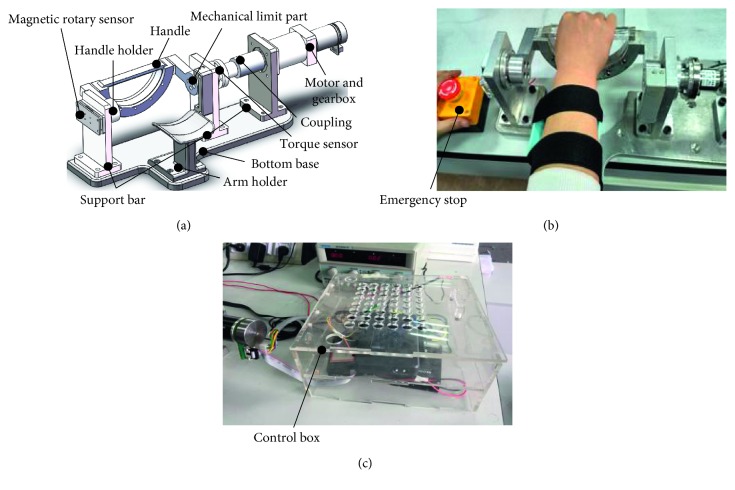
The WR*e*D system. (a) The 3D model, (b) its use on a healthy subject, and (c) the control box.

The prototype of WR*e*D has been built and reported in our previous work [24], as in Figure 1, where Figure 1(a) is the three-dimensional (3D) model of the device created in SolidWorks, Figure 1(b) shows its use on a healthy subject, and Figure 1(c) is the control box. The WR*e*D mainly consists of three components (the base module, the actuation module, and the arm-hand module). The base module acts as a foundation to support the actuation and the arm-hand module. It consists of the bottom base and three vertical support bars. The motor and two-stage reduction gear box comprise the actuation module. The arm-hand module consists of the arm holder, the handle holder, and the handle. The handle can be designed to be subject-specific based on the requirement. The mechanical limit part is set to avoid excessive rotation of the handle for safety reasons. Some bearings are also set to allow low friction motion transmission between the rotational axis and the base module. Before training, the human users will be instructed to make appropriate adjustments for their hands, including grabbing the handle though the handle holder, placing forearms on the arm holder, and fixing forearms with Velcro straps.

The electrical components of the WR*e*D consist of a DC motor (EC 45, Maxon), a motor driver (ESCON 50/5, Maxon), a static torque sensor (JNNT 25°Nm, Zhongwan), a magnetic rotary encoder (AS5048A, AMS), and an embedded controller (National Instruments myRIO-1900). The ESCON driver, myRIO controller, and the transmitter of the torque sensor are set in the control box as shown in Figure 1(c). The motor EC 45 nominally outputs 83.4°mNm, through the gear box with reduction ratio 1 : 300, and there is an estimated torque output of 25.02°Nm. With the consideration of the transmission efficiency being 0.7614, the WR*e*D can have a torque output of 19.05°Nm at the end effector (also the handle). The torque sensor is installed between the output shaft of the actuation module (through the coupling) and the handle holder, for measuring the torque of human and robot interaction. A magnetic rotary sensor is installed on the shaft of the handle holder for measuring the angular position of the human hand in real time. An emergency stop is also set to ensure training safety. Predefined data and those from the electrical components of the WR*e*D communicate with a computer through the embedded controller (myRIO-1900).

### 2.2. Control System

The trajectory tracking control of the WR*e*D is the basis of a variety of robot-assisted rehabilitation exercises. It can be directly used for passive training on patients with weak active motor ability. Tracking desired trajectories is not only a simple but also an effective method for rehabilitation applications [25]. Introducing compliance to trajectory tracking control can lead to enhanced training comfort and safety and also allows active engagement for better rehabilitation efficacy [26–28]. Compliance control is an important element during the interaction between patients and robots, since the interaction torque needs to be in a safe range. Meanwhile the position of the robot should also be controlled in the same way to minimize the follow error during the trajectory tracking [29]. Impedance control describes the dynamic relation between position and torque well and do not require accurate knowledge of the external environment when compared to the other methods, such as hybrid force and position control [17, 29]. Therefore, this technique has been broadly implemented in rehabilitation robots.

**Figure 2:**
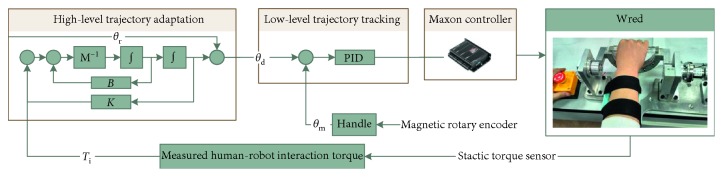
The control diagram of the WR*e*D.

Due to the large variability in human wrist characteristics and lack of accurate information of the environment, this paper proposes a two-level control system for trajectory tracking control of the WR*e*D with compliance adaption, as in Figure 2. In the low level, a closed loop system is used to achieve trajectory tracking by comparing desired trajectory from the high-level controller and actual trajectory measured from the magnetic rotary sensor. To allow trajectory generation with compliance adaptation, an admittance law is adopted in the high level. In general, the high-level controller dynamically adjusts the desired trajectory based on the feedback of the measured interaction torque and sends the angular position command to the low-level controller as an input signal.

In the high level, a reference trajectory can be defined by a physiotherapist or based on pilot tests, denoted as *θ*_r_(*t*). The admittance control law is proposed as in equation (1), where *θ*_r_(*t*) and *θ*_d_(*t*) represent the reference trajectory and the recalculated desired trajectory, respectively, and *T*_i_(*t*) is the patient-robot interaction torque, *B* and *K* respectively represent the damping and stiffness coefficients, and M is the inertia tensor. In this study, parameter *M* and *B* are assumed to be negligible due to low-velocity movement environment and low friction of the bearings. Thus, equation (2) can be derived. With the knowledge of the inertial property of the WR*e*D, the desired trajectory position *θ*_d_(*t*) can be derived. In this study, further in the middle level, the measured trajectory position *θ*_m_(*t*) is compared with *θ*_d_(*t*) for error in equation (3). Lastly, the error *e*(*t*) is input to the PID controller, and the required speed of the motor *v*(*t*) can be calculated according to equation (4) with well-tuned *K*_p_, *K*_i_, and *K*_d_. The parameter *K* in equations (1) and (2) is an indication of the device compliance. Specifically, the training with this controller can be considered to be completely passive when *K* is infinite, leading to *θ*_d_(*t*) approximately equal to *θ*_r_(*t*). With the variable *K* decreasing, the participant gets to be able to change the compliance of the WR*e*D with real-time human-robot interaction. The less the *K* is, the more compliance the device has, thus the participant can affect the predefined trajectory more easily.

(1) $$\begin{array}{c} T_{\text{i}}\left(t\right)=M\left(\ddot{\theta_{\text{d}}\left(t\right)}\;-\;\ddot{\theta_{\text{r}}\left(t\right)}\right)+B\left(\dot{\theta_{\text{d}}\left(t\right)}\;-\;\dot{\theta_{\text{r}}\left(t\right)}\right)+\;K\left(\theta_{\text{d}}\left(t\right)-\theta_{\text{r}}\left(t\right)\right), \end{array}$$

(2) $$\begin{array}{c} \theta_{\text{d}}\left(t\right)=\theta_{\text{r}}\left(t\right)+\frac{T_{\text{i}}\left(t\right)}{K}, \end{array}$$

(3) $$\begin{array}{c} e\left(t\right)=\theta_{\text{d}}\left(t\right)-\theta_{\text{m}}\left(t\right), \end{array}$$

(4) $$\begin{array}{c} v\left(t\right)=K_{\text{p}}e\left(t\right)+K_{\text{i}}\int_{0}^{t}e\left(t\right)\;dt+K_{\text{d}}\frac{de\left(t\right)}{dt}. \end{array}$$

### 2.3. Stability Analysis

To ensure the safety of the proposed compliance control strategy, it is essential to conduct stability analysis. Equation (1) can be transformed to equation (5), where *θ*_i_(*t*)=*θ*_r_(*t*) − *θ*_d_(*t*), *θ*_i_ is the position disturbance when the human wrist interacts with the WR*e*D. With the initial condition *θ*_i_(0)=0, $\dot{\theta_{\text{i}}}=0$, the transfer function (6) of the proposed compliance controller can be obtained through Laplace transformation. Thus equation (6) can be written as equation (7) to describe the relationship between the output and the input.

(5) $$\begin{array}{c} T_{\text{i}}\left(t\right)=-\left[M\ddot{\theta_{\text{i}}\left(t\right)}+B\dot{\theta_{\text{i}}\left(t\right)}+K\theta_{\text{i}}\left(t\right)\right], \end{array}$$

(6) $$\begin{array}{c} T_{i}\left(s\right)=-\left[Ms^{2}+Bs+K\right]X\left(s\right), \end{array}$$

(7) $$\begin{array}{c} G\left(s\right)=\frac{X\left(s\right)}{T_{\text{i}}\left(s\right)}=-\frac{1}{Ms^{2}+Bs+K}. \end{array}$$

The complex eigenvalue analysis method is a useful tool for analyzing the stability of control systems, which requires the calculation of the system's complex eigenvalues. The corresponding characteristic equation of equation (7) can be written as follows:

(8) $$\begin{array}{c} Ms^{2}+Bs+K=0. \end{array}$$

An eigenvalue of equation (8) is in complex form of *α*+*jω*, where *α* is the real part of *s*, which indicates the stability of the system and *ω* is the imaginary part of *s*, which indicates the modal frequency [30]. The eigenvalues of equation (8) can be obtained as follows:

(9) $$\begin{array}{c} s=\frac{-B\pm \sqrt{B^{2}-4MK}}{2M}. \end{array}$$

With *B* > 0 and *K* > 0, it is obviously seen that the system is stable with both eigenvalues in the left-half of the complex plane. The stability level depends on the real parts of eigenvalues. Therefore, the controller parameter design is critical to achieve a stable control system.

**Figure 3:**
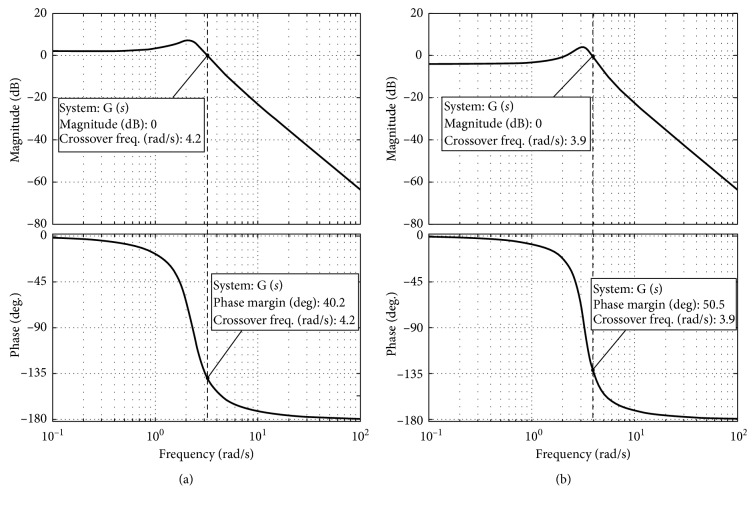
The Bode diagrams of the proposed compliance controller with two different configurations: (a) *M* = 0.15, *B* = 0.2, and *K* = 0.8; (b) *M* = 0.15, *B* = 0.2, and *K* = 1.6.

To visualize the stability status, the Bode diagram method is adopted. To conduct stability analysis with different compliance levels, the variable *K* was set with different values. Pretests were conducted, and it was found that when the robot compliance has significant differences when *K* is 0.8 or 1.6. A sequence of pretests was also carried out with the different values of *M* and *B*. From the Bode diagrams, the phase margin plots can be obviously presented when *M* = 0.15 and *B* = 0.2. By using this method, the controller parameters are assumed with two groups according to the compliance difference: *M* = 0.15, *B* = 0.2, and *K* = 0.8 and *M* = 0.15, *B* = 0.2, and *K* = 1.6. The Bode diagrams of the proposed compliance controller are presented in Figures 3(a) and 3(b). In the case of *K* = 0.8, it shows the phase margin *γ*=40.2° with the gain crossover frequency *ω*_C_=4.2  rad/s. While in the case of *K* = 1.6, the phase margin is *γ*=50.5° with the gain crossover frequency *ω*_*C*_=3.9  rad/s. Since both of the phase margin plots do not cross with −180°, the gain margins become infinity. According to the Bode stability criterion [31], the minimum phase system is stable with the phase margin *γ* > 0 and the phase margin *h* > 1. Back to the compliance controller part, it is a minimum phase system since both eigenvalues are in the left-half of the complex plane. Therefore, the compliance controller part is stable.

### 2.4. Participants and Training Protocol

To preliminarily evaluate the performance of the proposed control system on the WR*e*D, a healthy subject volunteered to participate in the test in a lab environment. The participant is male with the age of 27 years old, 170 cm height, and 65 kg body weight. Before the training, the actual range of motion (ROM) of the wrist joint was measured for safety. The healthy subject followed the training instructions well and had no confusion in using the WR*e*D. The test was under the supervision of experienced clinical personnel and engineers.

Passive training has been demonstrated as an effective way to induce quick recovery and expand joint ROM [32, 33]. Trajectory tracking is a conventional method used to investigate the performance of passive motions. In this study, the training trajectory is set as a sine wave piecewise function, as in equation (5), which allows the training to be slow at wrist flexion/extension limited position for comfort and safety. Based on the measurements by using traditional goniometers, the participant has the wrist flexion up to 80° and the wrist extension up to 65°. This is consistent with published data of normal wrist range of motion 0–80° for flexion and 0–70° for extension [23]. Here, it is defined the wrist flexion for negative and extension for positive. Thus, the parameter *A*_ext_ in equation (5) is set as 65° and *A*_fle_ as −80°. The frequency *f* is set to be 0.05 Hz. In equation (4), the parameters *K*_p_, *K*_i_, and *K*_d_ are tuned by using Cohen Coon method [34] to be 0.3, 0.6, and 0 for tracking desired trajectory *θ*_d_(*t*).

(10) $$\begin{array}{c} \theta_{\text{r}}\left(t\right)=\left\{\begin{array}{cc} A_{\text{ext}}\sin\left(2\;*\text{ pi }*\;f\;*\;t\right), & \text{when}\;\;\theta_{\text{m}}\left(t\right)\geq 0,\\ A_{\text{fle}}\sin\left(2\;*\text{ pi }*\;f\;*\;t\right), & \text{when}\;\;\theta_{\text{m}}\left(t\right)<0. \end{array}\right. \end{array}$$

To validate the trajectory tracking responses and whether the proposed controller is capable of changing the compliance of the WR*e*D, four experiments were conducted with each for 10 cycles. Experiment 1 was conducted with zero load using only trajectory tracking control (without trajectory adaptation). By the same control method, Experiment 2 was conducted with the hand of the participant on. Experiments 3 and 4 were conducted to check whether the proposed method is capable of changing the compliance of the WR*e*D, with the parameter *K* being 0.8 and 1.6, respectively. During these experiments, the healthy subject was verbally encouraged to relax his wrist during passive training.

## 3. Experimental Results

**Figure 4:**
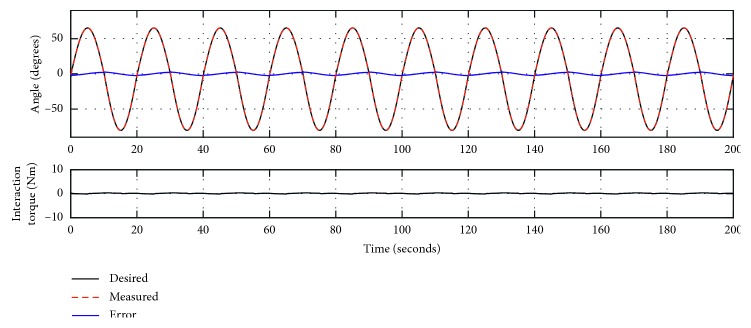
The trajectory tracking responses of Experiment 1 (zero load).

The trajectory tracking response of Experiment 1 is presented in Figure 4 by using MATLAB R2016a, where the dark line sine wave is the desired trajectory, the dotted red line is the measured trajectory, the blue line is the error as in equation (3), and the dark line in the bottom plot is the measured torque from the torque sensor. Without external load, the desired trajectory compares well with the desired trajectory. To allow quantitative analysis of the trajectory tracking performance under this control method, the statistical results are given in Table 1, with the error ranging from −2.998° to 3.074°, the root mean square deviation (RMSD) being 1.5682°, and the normalized root mean square deviation (NRMSD) being 1.08%. Figure 4 also shows only a slight variation of the interaction torque throughout the training. This is due to the lack of human-robot interaction, and the small variation is caused by friction between the arm-hand module and the actuation module. As statistical results in Table 1, the mean of absolute torque value (MATV) is only 0.152°Nm.

**Figure 5:**
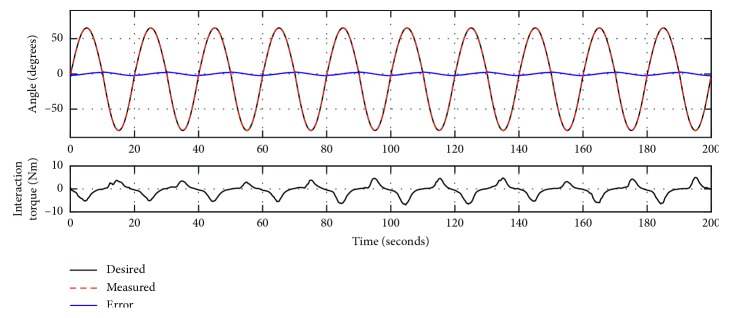
The trajectory tracking responses of Experiment 2 (passive training).

Figure 5 presents the trajectory tracking response of Experiment 2, using the same control method as that in Experiment 1. During the whole process, the participant was encouraged to relax and did not apply intentional active force on the handle. To allow quantitative comparison with Experiment 1, statistical results are also given in Table 1. The trajectory tracking error ranges from −3.054° to 3.110°, the RMSD value is 1.5767°, and the NRMSD value is 1.08%. By comparing with the interaction torque presented in Figure 4 and Table 1, there is more obvious torque variation during each cycle of training, with the MATV value being 1.825°Nm. This is caused by the resistance when the wrist is at limited joint position. Experiments 1 and 2 both show satisfactory trajectory tracking performance, with the NRMSD value being 1.08%.

**Figure 6:**
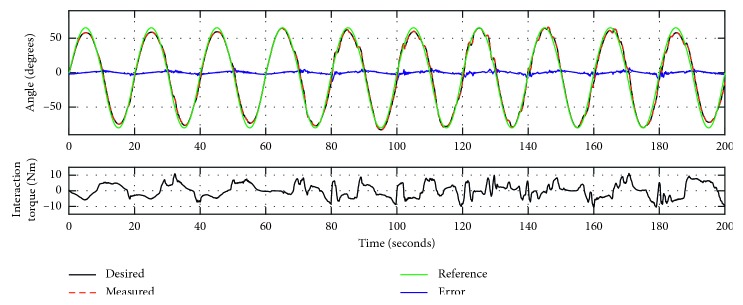
The trajectory tracking responses of Experiment 3 (*K* = 0.8).

**Figure 7:**
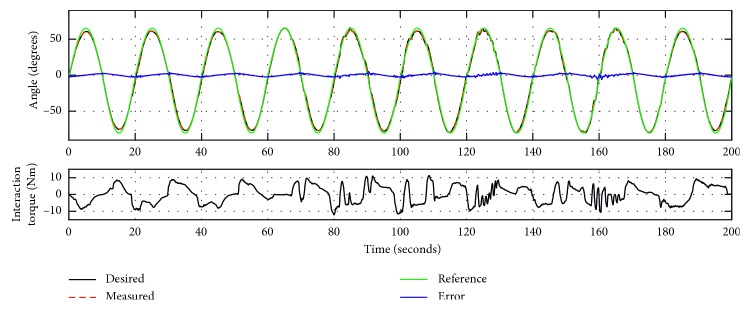
The trajectory tracking responses of Experiment 4 (*K* = 1.6).

To evaluate the proposed control method, especially the high-level trajectory adaptation method to adjust the compliance of the WR*e*D, Experiments 3 and 4 were conducted with the same participant. The parameter *K* in equation (2) represents the stiffness coefficient of the WR*e*D. Experiment 3 has the *K* value of 0.8, and trajectory tracking response is presented in Figure 6. In the top plot, the dark line is the desired trajectory after adaptation from the reference sine wave trajectory (plotted in green), the dotted red line is the measured trajectory, and the blue line is the error of the trajectory tracking. As summarized in Table 1, the trajectory tracking error ranges from −12.784° to 7.998°, the RMSD value is 2.0879°, and the NRMSD value is 1.4%. Experiment 4 has the *K* value of 1.6, and trajectory tracking response is shown in Figure 7. As summarized in Table 1, the trajectory tracking error ranges from −6.081° to 5.211°, the RMSD is 1.6211°, and the NRMSD is 1.12%. It was found that the desired trajectory was adapted based on the interaction torque with respect to the reference trajectory.

Furthermore, to analyze the experimental results presented in Figures 6 and 7 and Table 1, while Experiment 3 has a smaller interaction torque with the MATV of 3.669°Nm, it presents a larger NRMSD^*∗*^ value (4.13%) of the trajectory adaptation. This demonstrates that the control method with *K* value being 0.8 has more compliance with respect to Experiment 4 (*K* = 1.6). As the stiffness coefficient of the WR*e*D increases, the training can be considered to approach to passive mode, similar to Experiment 2. In terms of the trajectory tracking accuracy of Experiments 3 and 4, the latter has a better tracking performance than the former, with NRMSD values being 1.12% and 1.4%, respectively. This finding is reasonable since the WR*e*D system with more compliance is more susceptible to external disturbance, which is also reflected in Table 1 with the MinError of Experiment 3 up to 12.784°. The data for Experiment 1, 2, 3, and 4 are included within the supplementary information files to support the findings of this study.

## 4. Discussion and Conclusion

This paper presents the development of the WR*e*D and a compliance control method for comfortable and safe human-robot interaction. By contrast with existing wrist rehabilitation robots [15, 18, 22], the WR*e*D is portable for use in hospital or home environment due to its reconfigurable structure design. In this study, the WR*e*D is configured for the rehabilitation training of wrist flexion and extension, as shown in Figure 1, since wrist flexion and extension are worth 70% of total wrist function [35], and training therapy along this direction is more commonly used than that of radial and ulnar deviation. It is worth mentioning that the WR*e*D can be easily reconfigured for training of wrist radial and ulnar deviation by rotating the handle to a vertical posture along the handle holder. However, this is not the focus of this study. More importantly, this has no effect on the experiment design as well as the feasibility evaluation of the proposed control method. This study presents experimental validation of the proposed interactive compliance control with satisfactory performance, summarized in Table 1. While tests were conducted only with wrist flexion and extension, it is reasonable to predict that the proposed method will also apply to the training of wrist radial and ulnar deviation.

Human-robot interactive tasks cannot be handled by pure motion control that generally rejects forces exerted by human users as disturbances. The impedance control scheme is mostly considered as the basis of interactive robotic training. It has been widely adopted in rehabilitation robots [29, 36] to obtain user comfort and enhance the training safety. There are two ways of implementing impedance control based on the controller causality: impedance control (force- or torque-based method) and admittance control (position-based method) [37–39]. While both implementations were referred to as impedance control by Hogan [40], we make a distinction since this is highly relevant to the work presented in this study. The impedance controller takes a displacement as input and reacts with a force, while in an admittance control mode, the controller is an admittance and the manipulator is an impedance [28]. The interactive compliance control of the WR*e*D was achieved based on an admittance law due to the availability of direct measurement of the interaction torque. Under admittance mode, the WR*e*D deviates from the reference trajectory in the presence of patient-robot interaction but is otherwise following the reference trajectory.

Experiments were conducted by implementing the trajectory tracking combined with compliance control. As the statistical results in Table 1, the proposed compliance control method has a satisfactory trajectory tracking accuracy, with all NRMSD values no greater than 1.4%. The stability of the compliance controller was analyzed by calculating the complex eigenvalues and also has been validated with the sufficient phase margin and gain margin according to the Bode stability criteria. Different stiffness coefficients were manually set to evaluate the effect on the compliance of the presented control method. The introduction of the compliance control method can make robot-assisted wrist rehabilitation training more comfortable and safer by avoiding excessive interaction torque on human wrists.

While experiments have been conducted with satisfactory compliance and trajectory tracking performance, this study suffers from some limitations. First, the developed WR*e*D can be further improved for clinical applications. Some measures should be taken to address the issue of misalignment between the device and the human wrist and to comfortably fix human arms preventing relative movements. This may have affected the measurement of the position and interaction torque of human wrists. Second, to achieve better control performance, the inertial property of the WR*e*D should be considered in equation (1), as well as the inertial property of human hands. Third, this study has only one participant for such a preliminary test; more subjects with hand disabilities should be encouraged and recruited in future tests.

Future work will focus on the improvement of the WR*e*D in terms of its functionality and clinical evaluation. Mechanically, we will consider designing different handles for use on different patients, addressing the misalignment issue, also adding another actuation module for multiple dimension training. For applications, this device can be used for not only rehabilitation training, but also assessment purpose in measuring wrist stiffness and ranges of motion. Its efficacy for assessment will be investigated next. Directly following this study, more research is needed to improve the robustness of the proposed interactive compliance control scheme to external disturbances and involuntary human-robot interaction. Future work will also consider proposing a high-level algorithm to enable automatic *K* value tuning.

To summarize, this paper proposes an interactive compliance control scheme on the WR*e*D for enhanced training safety and efficacy. This control system consists of a low-level trajectory tracking loop and a high-level admittance loop. Experiments were conducted with zero load and human interaction, respectively. Satisfactory trajectory tracking responses were obtained, with the NRMSD values being 1.08% with zero load and the NRMSD values no greater than 1.4% with real-time disturbance and interaction from the human user. The interactive compliance can be adjusted in subject specific by setting different stiffness coefficients of the control system. Results demonstrate that such an interactive compliance control method can adaptively adjust the range of training motions and encourage human users' active engagement. These findings suggest that the proposed control method of the WR*e*D has great potentials for clinical applications due to enhanced training safety and efficacy.

## Figures and Tables

**Table 1:** Experimental results of the tracking responses of all experiments.

			MinError (°)	MaxError (°)	RMSD (°)	NRMSD (%)	MATV (Nm)	NRMSD^*∗*^ (%)
Zero load		Experiment 1	−2.998	3.074	1.5682	1.08	0.152	NA
Passive training		Experiment 2	−3.054	3.110	1.5767	1.08	1.825	NA
Interaction training	*K* = 0.8	Experiment 3	−12.784	7.998	2.0879	1.4	3.669	4.13
	*K* = 1.6	Experiment 4	−6.081	5.211	1.6211	1.12	4.446	2.24

*Note.* MinError is the maximum tracking error in negative direction, which is numerically minimum; MaxError is the maximum tracking error in positive direction, which is numerically maximum. RMSD: root mean square deviation; NRMSD: normalized root mean square deviation; MATV: mean of absolute torque value; NRMSD^*∗*^ represents the NRMSD value of the adapted desired trajectory *θ*_d_(*t*) with respect to the reference trajectory *θ*_r_(*t*); NA: not applicable.

## Data Availability

The data for Experiment 1, 2, 3, and 4 are included within the supplementary information files to support the findings of this study. Also, we share the original data as the supplementary information files.

## Supplementary Materials

Supplementary Materials The original data of Experiment 1 to Experiment 4 are included. The data file of each experiment contains four columns named Time (second), Desired Angle (degree), Interaction Torque (Nm), and Measured Angle (degree). Figures 4–7 were derived from the original data of Experiment 1 to Experiment 4 by using MATLAB. Below are the descriptions of the four experiments: Experiment 1: the trajectory tracking responses with zero load. Experiment 2: the trajectory tracking responses with passive training. Experiment 3: the trajectory tracking responses with *K* = 0.8. Experiment 4: the trajectory tracking responses with *K* = 1.6.

## References

[1] Johnston S. C., Mendis S., Mathers C. D. (2009). Global variation in stroke burden and mortality: estimates from monitoring, surveillance, and modelling. *The Lancet Neurology*.

[2] Mozaffafian D., Benjamin E. J., Go A. S. (2015). Heart disease and stroke statistics—2015 Update. *Circulation*.

[3] “Facts about stroke in New Zealand. https://www.stroke.org.nz/facts-and-faqs.

[4] Turley M., Ministry of Health (2013). *Health Loss in New Zealand: a Report from the New Zealand Burden of Diseases, Injuries and Risk Factors Study, 2006–2016*.

[5] Caplan L. R. (2006). *Stroke*.

[6] Lum P. S., Burgar C. G., Shor P. C., Majmundar M., Van der Loos M. (2002). Robot-assisted movement training compared with conventional therapy techniques for the rehabilitation of upper-limb motor function after stroke. *Archives of Physical Medicine and Rehabilitation*.

[7] Loureiro R., Amirabdollahian F., Topping M., Driessen B., Harwin W. (2003). Upper limb robot mediated stroke therapy—GENTLE/s approach. *Autonomous Robots*.

[8] Kleim J. A., Jones T. A., Schallert T. (2003). Motor enrichment and the induction of plasticity before or after brain injury. *Neurochemical Research*.

[9] Chang Y.-J., Han W.-Y., Tsai Y.-C. (2013). A Kinect-based upper limb rehabilitation system to assist people with cerebral palsy. *Research in Developmental Disabilities*.

[10] Pehlivan A. U., Lee S., O’Malley M. K. Mechanical design of RiceWrist-S: a forearm-wrist exoskeleton for stroke and spinal cord injury rehabilitation.

[11] Krebs H. I., Palazzolo J. J., Dipietro L. (2003). Rehabilitation robotics: performance-based progressive robot-assisted therapy. *Autonomous Robots*.

[12] Kwakkel G., Kollen B. J., Krebs H. I. (2008). Effects of robot-assisted therapy on upper limb recovery after stroke: a systematic review. *Neurorehabilitation and Neural Repair*.

[13] Stein J., Krebs H. I., Frontera W. R., Fasoli S. E., Hughes R., Hogan N. (2004). Comparison of two techniques of robot-aided upper limb exercise training after stroke. *American Journal of Physical Medicine & Rehabilitation*.

[14] Liao W.-W., Wu C.-Y., Hsieh Y.-W., Lin K.-C., Chang W.-Y. (2012). Effects of robot-assisted upper limb rehabilitation on daily function and real-world arm activity in patients with chronic stroke: a randomized controlled trial. *Clinical Rehabilitation*.

[15] Oblak J., Cikajlo I., Matjacic Z. (2010). Universal haptic drive: a robot for arm and wrist rehabilitation. *IEEE Transactions on Neural Systems & Rehabilitation Engineering*.

[16] Mazzoleni S., Carrozza M. C., Sale P., Franceschini M., Posteraro F., Tiboni M. Effects of upper limb robot-assisted therapy on motor recovery of subacute stroke patients: a kinematic approach.

[17] Volpe B. T., Krebs H. I., Hogan N., Edelsteinn L., Diels C. M., Aisen M. L. (1999). Robot training enhanced motor outcome in patients with stroke maintained over 3 years. *Neurology*.

[18] Krebs H. I., Volpe B. T., Williams D. (2007). Robot-aided neurorehabilitation: a robot for wrist rehabilitation. *IEEE Transactions on Neural Systems and Rehabilitation Engineering*.

[19] Faghihi A., Haghpanah S. A., Farahmand F., Jafari M. Design and fabrication of a robot for neurorehabilitation; smart robo wrist.

[20] Toth A., Fazekas G., Arz G., Jurak M., Horvath M. Passive robotic movement therapy of the spastic hemiparetic arm with REHAROB: report of the first clinical test and the follow-up system improvement.

[21] Colombo R., Pisano F., Micera S. (2005). Robotic techniques for upper limb evaluation and rehabilitation of stroke patients. *IEEE Transactions on Neural Systems and Rehabilitation Engineering*.

[22] Khor K. X., Chin P. J. H., Hisyam A. R., Yeong C. F., Narayanan A. L. T., Su E. L. M. Development of CR2-Haptic: a compact and portable rehabilitation robot for wrist and forearm training.

[23] Youm Y., Flatt A. E. (1984). Design of a total wrist prosthesis. *Annals of Biomedical Engineering*.

[24] Zhang M., Xu D., Sun Y. Development of a reconfigurable wrist rehabilitation device with an adaptive forearm holder.

[25] Proietti T., Crocher V., Roby-Brami A., Jarrassé N. (2016). Upper-limb robotic exoskeletons for neurorehabilitation: a review on control strategies. *IEEE Reviews in Biomedical Engineering*.

[26] Zhang M., Davies T. C., Xie S. (2013). Effectiveness of robot-assisted therapy on ankle rehabilitation–a systematic review. *Journal of Neuro Engineering and Rehabilitation*.

[27] Yu H., Huang S., Chen G., Pan Y., Guo Z. (2015). Human–robot interaction control of rehabilitation robots with series elastic actuators. *IEEE Transactions on Robotics*.

[28] Zhang M., Xie S. Q., Li X. (2017). Adaptive patient-cooperative control of a compliant ankle rehabilitation robot (CARR) with enhanced training safety. *IEEE Transactions on Industrial Electronics*.

[29] Tsoi Y. H., Xie S. Q. Impedance control of ankle rehabilitation robot.

[30] Wang X., Mo J. L., Ouyang H. (2015). Squeal noise of friction material with groove-textured surface: an experimental and numerical analysis. *Journal of Tribology*.

[31] Coughanowr D. R., Koppel L. B. (1991). *Process Systems Analysis and Control*.

[32] Salter B. R., Field P. (1960). The effects of continuous compression on living articular cartilage: an experimental investigation. *Journal of Bone & Joint Surgery*.

[33] O’Driscoll S. W., Giori N. J. (2000). Continuous passive motion (CPM): theory and principles of clinical application. *Journal of Rehabilitation Research & Development*.

[34] Zawawi M. Z. F. B. M., Elamvazuthi I., Aziz A. B. A., Daud S. A. Comparison of PID and fuzzy logic controller for DC servo motor in the development of lower extremity exoskeleton for rehabilitation.

[35] Swanson A. B., Hagert C. G., Swanson G. D. (1983). Evaluation of impairment of hand function. *Journal of Hand Surgery*.

[36] Yang Y., Wang L., Tong J., Zhang L. Arm rehabilitation robot impedance control and experimentation.

[37] Lawrence D. A. Impedance control stability properties in common implementations.

[38] Ott C., Mukherjee R., Nakamura Y. Unified impedance and admittance control.

[39] Ott C., Nakamura Y. Base force/torque sensing for position based Cartesian impedance control.

[40] Hogan N. Impedance control: an approach to manipulation.

